# Field transcriptome revealed a novel relationship between nitrate transport and flowering in Japanese beech

**DOI:** 10.1038/s41598-019-39608-1

**Published:** 2019-03-13

**Authors:** Akiko Satake, Kazutaka Kawatsu, Kosuke Teshima, Daisuke Kabeya, Qingmin Han

**Affiliations:** 1 Department of Biology, Faculty of Science, Kyushu University, 819-0395 Fukuoka, Japan; 2 Graduate School of Life Sciences, Tohoku University, 980-8578 Sendai, Japan; 3 Department of Plant Ecology, Forestry and Forest Products Research Institute (FFPRI), 305-8687 Tsukuba, Japan

## Abstract

Recent advances in molecular and genetic studies about flowering time control have been increasingly available to elucidate the physiological mechanism underlying masting, the intermittent and synchronized production of a large amount of flowers and seeds in plant populations. To identify unexplored developmental and physiological processes associated with masting, genome-wide transcriptome analysis is a promising tool, but such analyses have yet to be performed. We established a field transcriptome using a typical masting species, Japanese beech (*Fagus crenata* Blume), over two years, and analyzed the data using a nonlinear time-series analysis called convergent cross mapping. Our field transcriptome was found to undergo numerous changes depending on the status of floral induction and season. An integrated approach of high-throughput transcriptomics and causal inference was successful at detecting novel causal regulatory relationships between nitrate transport and florigen synthesis/transport in a forest tree species. The synergistic activation of nitrate transport and floral transition could be adaptive to simultaneously satisfy floral transition at the appropriate timing and the nitrogen demand needed for flower formation.

## Introduction

The intermittent and synchronized production of large amount of flowers and seeds, called masting or mast seeding^1–3^, is a widespread phenomenon among plant species^4^. In commercially important fruit species, masting is known as biennial or alternate bearing—the alternation of high- and low-crop years^5^. In natural ecosystems, the drastic increase in seed production in mast years has a considerable effect on seedling recruitment^6^ and animal populations that feed on seeds^7–9^. Thus, understanding the mechanism of masting has long been a major issue in ecological studies. Previous ecological and theoretical studies demonstrated that external environmental factors and endogenous nutrient status influence the induction of masting^10–12^. However, the detailed physiological mechanisms underlying masting have remained elusive.

Applying recent advances in molecular and genetic studies on flowering time control to masting species has become increasingly useful to unravel the mechanism underlying masting^13–15^. In *Arabidopsis thaliana*, environmental and developmental signals perceived in different flowering pathways ultimately converge on a small number of genes called floral pathway integrators^16–18^, such as *FLOWERING LOCUS T* (*FT*)^19,20^. Recent studies using *FT* expression levels as a molecular marker of flower masting have discovered the key role of nitrogen in the induction of flowering in a typical masting species, Japanese beech (*Fagus crenata* Blume)^14^. Extending the target gene analyses to genome-wide transcriptome analysis is promising to identify unexplored developmental and physiological processes associated with masting.

**Figure 1:**
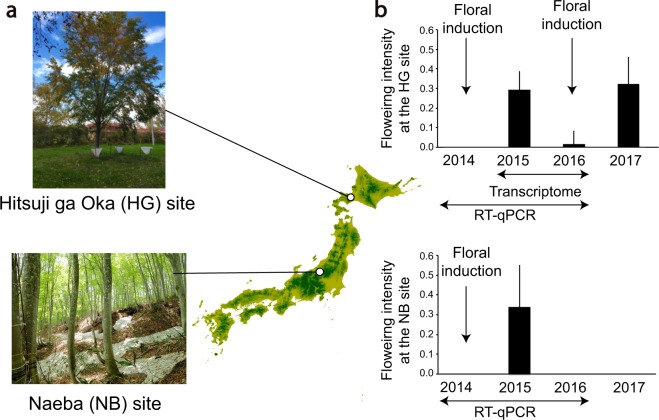
Locations of two study sites and between-year fluctuation of flowering intensity. (**a**) Locations of the HG and NB study sites and Japanese beech used in this study. (**b**) Flowering intensity calculated as the average of the proportion of buds which are reproductive at the HG site (mean ± s.d. of three individuals) and at the NB site (mean ± s.d. of six individuals). When the proportion of buds which are reproductive was greater than 0.4, the year prior to anthesis was assigned as a floral induction year for the tree because floral induction occurs one year prior to anthesis; otherwise, it was assigned as a non-induction year. At the HG site, floral induction occurred in two trees, HG1 and HG2, both in 2014 and 2016. At the NB site, floral induction occurred in three trees, NB1, NB5, and NB6 in 2014. Horizontal arrows indicate the census period for field transcriptome and RT-qPCR, respectively. Vertical arrows stand for the year when floral induction occurred.

This is the first study to establish a field transcriptome using masting species to detect novel causal relationships between floral induction and potential physiological processes crucial for masting. We performed DNA microarray analysis using leaf and bud samples collected monthly during the growing season from three individual Japanese beech in Hokkaido (HG site; Fig. 1a), Japan, over two years. Field transcriptome data were analyzed using a nonlinear time-series analysis called convergent cross-mapping (CCM)^21^. CCM has been increasingly used in ecological studies to dissect complex interaction networks between species in ecological communities^22,23^. A similar method, called Cross Map Smoothness, has also been used for causal inference of the gene regulatory network^24^. In non-linear systems, visual inspection or simple pairwise correlations between variables can be misleading about the nature of causality. In such cases, CCM is able to robustly detect causality which may not be detectable by methods which fail to allow for the non-linearity^21^. The causal relationship identified from transcriptome data obtained in this study was confirmed using independent data from six individual trees at Mt. Naeba (NB site; Fig. 1a) in the center of Honshu, Japan. Here, we show that combining high-throughput transcriptomic analysis with CCM time-series analysis is a powerful framework to detect the novel relationship between nitrate transport and flowering.

## Results

**Figure 2:**
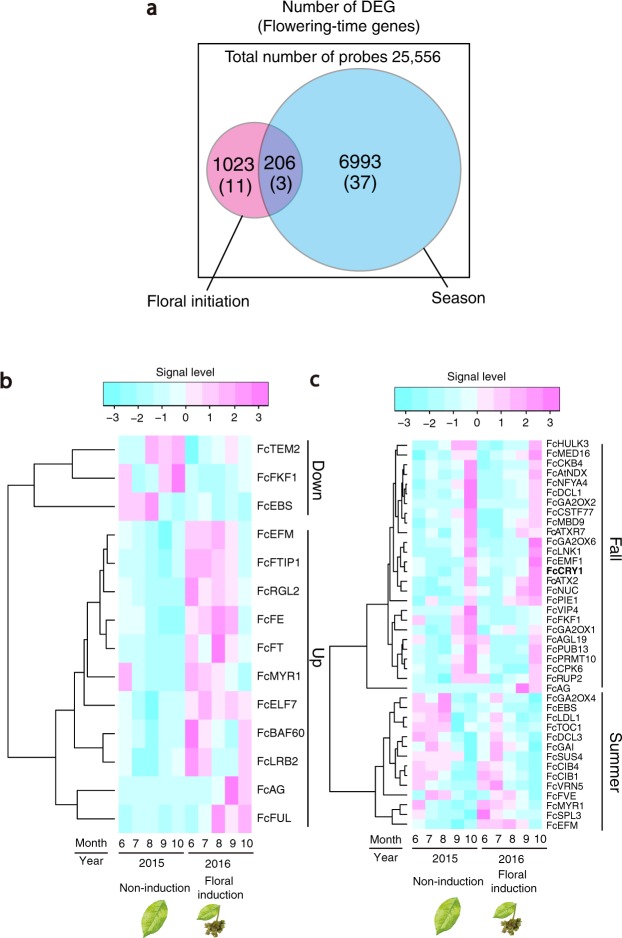
Venn diagram of differentially expressed genes (DEGs) and heat map of flowering-time genes in *F*. *crenata*. (**a**) Venn diagram of DEGs between both floral induction status and season. (**b**) Heat map of differentially expressed flowering-time genes between induction and non-induction years of flowering. The dendrogram of the 14 classification probe sets is shown on the left. (**c**) Heat map of differentially expressed flowering-time genes between summer and fall. A dendrogram of the 30 classification probe sets is shown on the left. *FcCRY1* was highlighted by bold. To draw the heat map, mean signal levels over three individuals were used for each gene. Note that floral induction did not occur in the individual HG3 in 2016.

In Japanese beech, flower induction occurs in early summer in the year prior to anthesis. After winter dormancy, buds break and flowers bloom in spring. During the two-year monitoring of the transcriptome at the HG site (2015–2016), floral induction occurred only in 2016, which was followed by anthesis the next spring (Fig. 1b). Depending on the status of floral induction, in total, 1,229 probes (genes) were differentially expressed (two-way ANOVA, p-value < 0.01; Fig. 2a). Approximately six times more probes (7,199 probes) were detected as differentially expressed genes (DEGs) between summer and fall, among which 206 probes overlapped between these two groups (two-way ANOVA, p-value < 0.01; Fig. 2a).

Among the 1,229 DEGs between years with and without floral induction, 534 probes had a unique Blast hit against genes of *Arabidopsis thaliana* (Supplementary Table S1), among which 14 were identified as flowering-time genes in *F*. *crenata* (Fig. 2a). A total of 11 out of these 14 DEGs were upregulated in the years of floral induction prior to anthesis and the other 3 genes were downregulated (Fig. 2b). Among the 11 upregulated genes, *FcFT* was expressed in August in the years of floral induction that was followed by peaks of *AGAMOUS* (*FcAG*) and *FRUITFUL* (*FcFUL*) in fall (Fig. 2b). *AG* and *FUL* are MADS-box transcription factors associated with floral organ identity specification^25–27^. Activation of these genes after the peak of *FcFT* is consistent with the finding in *A*. *thaliana* that these genes act downstream of *FT*^18^. Genes involved in FT transport, *FT-INTERACTING PROTEIN* (*FcFTIP1*)^28^ and *FcFE*, a phloem-specific Myb-related protein^29^, were also activated in the year of floral induciton in Japanese beech (Fig. 2b), suggesting that FT synthesis and transport occur synergistically.

We also identified three genes, *TEMPANILLO 2* (*FcTEM2*), *EARLY BOLTING IN SHORT DAYS* (*FcEBS*), and *FLAVIN-BINDING*, *KELCH REPEAT*, *F-BOX 1* (*FcFKF1*) that were downregulated in the years of floral induction (Fig. 2b). *TEM2* and *EBS* are known as floral repressors in *A*. *thaliana*. *TEM2* directly binds to *FT* to suppress its transcription^30^. *EBS* also directly binds to *FT* and is required to maintain an inactive chromatin configuration by preventing high levels of H3 acetylation in regulatory regions^31^. Downregulation of these floral repressors coincided with the upregulation of *FcFT*, implying that a similar regulatory relationship among *FcFT*, *FcTEM*, and *FcEBS* exists in *F*. *crenata*. In contrast, *FKF1* has been reported to promote flowering by stabilizing *CONSTANS* protein that upregulates *FT* in *A*. *thaliana*^32^. Activation of *FcFT* even at the low expression level of *FcFKF1* suggests that *FcFKF1* would not be crucial for floral induciton in *F*. *crenata*.

Overall, 40 genes, corresponding to about 30% of the total of 153 flowering-time genes identified in *F*. *crenata* (Supplementary Table S2), were differentially expressed between summer and fall (Fig. 2c). Among 25 genes that were upregulated in fall (two-way ANOVA, p-value < 0.01; Fig. 2c), *FcCRY1* that encodes a protein associated with a flavin chromophore (FAD) absorbing blue/UV-A revealed elevated expression in October prior to leaf shedding (Fig. 2c). Most of the fall-upregulated genes showed similar sharp peaks in expression in October (Fig. 2c). Three genes (*FcEBS*, *FcMYR1*, *FcEFM*) were differentially expressed depending on both floral induction and season (Fig. 2a).

To identify novel relationships between flowering and other functional pathways, we first performed functional categorization of 520 annotated DEGs between years of induction and non-induction of flowering based on Gene Ontology (GO) terms^33^. Our GO analysis revealed that genes related to low-affinity nitrate transport were enriched in both the biological process and molecular function categories (Supplementary Figs S1 and S2). In addition to low-affinity nitrate transport, genes related to the regulation of transcription accounted for a substantial fraction of the total in the biological process category. Genes were also enriched for the development term, including in the subcategories of embryo development ending in seed dormancy and plant ovule development, and responses to external signals including response to fungus and nematode in the biological process category (Supplementary Fig. S1). These findings suggested a relationship among floral induction, development, and sensitivity to environmental signals.

**Figure 3:**
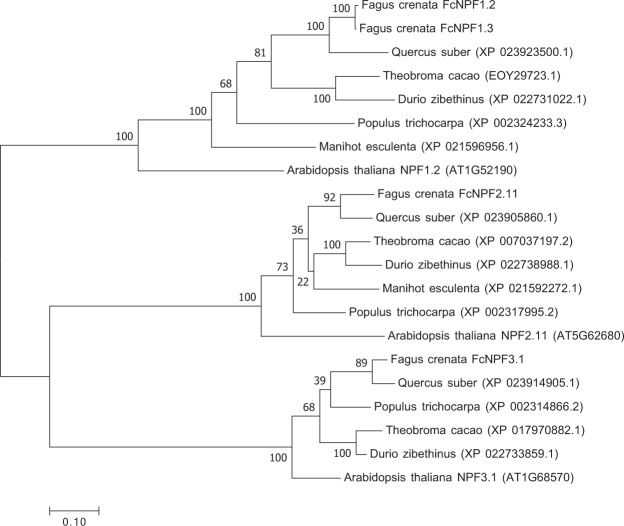
Phylogenetic relationships of NPF proteins in *F*. *crenata* and *A*. *thaliana*. The phylogenetic tree was created using the Maximum Likelihood method based of the JTT matrix-based model in MEGA7. All positions containing gaps and missing data were eliminated. The percentages of replicate trees in which the associated taxa clustered together in the bootstrap test (1000 replicates) are shown next to the branches. The numbers in parentheses are DDBJ and GenBank accession numbers (http://www.ddbj.nig.ac.jp).

Because a previous study identified the key role of nitrogen in floral induction in Japanese beech^14^, we further explored the details of the probes found to be associated with low-affinity nitrate transport in GO analysis. We annotated the two probes found to be associated with low-affinity nitrate transport in GO analysis using BLASTN searches against National Center for Biotechnology Information (NCBI) nucleotide nonredundant protein sequences. The two probes had a unique hit to the same gene, NRT1/PTR FAMILY 1.2 (Nitrate Transporter1/Peptide Transporter Family; NPF1.2/NRT1.11), in diverse plant species including *Quercus suber* [protein sequence similarity (PSS) = 82%], *Populus trichocarpa* [PSS = 65%], *Vitis vinifera* [PSS = 61%], and *A*. *thaliana* [PSS = 53%]. The gene trees based on amino acid sequences from diverse plant species showed that our isolated putative gene is found predominantly in the NPF1.2/NRT1.11 (hereafter NPF1.2) clade (Fig. 3). These results indicate that our putative gene is a homolog of NPF1.2. NPF family members encode nitrate or peptides transporters, some of which can also transport hormones in *A*. *thaliana*^34,35^. In *A*. *thaliana*, NPF1.2 has been reported to be localized in the companion cells of major veins and to be involved in the transfer of xylem-borne nitrate to the phloem^36^, contributing to the remobilization of nitrate from source to sink tissues^37^.

**Figure 4:**
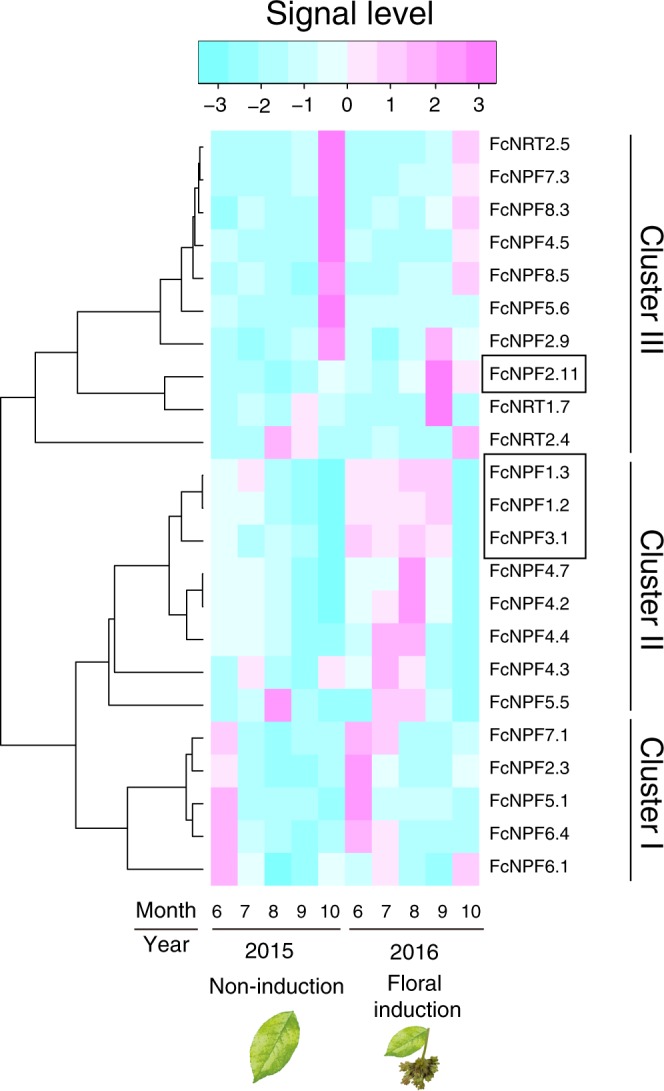
Heat map of different NPF and NRT2 genes in *F*. *crenata*. Heat map of NPF and NRT2 genes in Japanese beech. DEGs between years with and without floral induction were highlighted by squares. To draw the heat map, mean signal levels over three individuals were used for each gene. Note that floral induction did not occur in the individual HG3 in 2016.

Our transcriptome data included 20 and 2 other probes that were predicted to be NPF and nitrate transporter 2 (NRT2) family, respectively (Supplementary Table S3; Supplementary Fig. S2). These nitrate transporter genes were expressed sequentially over the course of a year (Fig. 4). Specifically, genes in cluster 1 were highly expressed in early summer (Fig. 4). Genes in cluster 2 were expressed in mid-summer, which was followed by the expression of genes in cluster 3 in fall (Fig. 4). Among these 22 genes, *FcNPF1*.*3* and *FcNPF3*.*1* in cluster 2 and *FcNPF2*.*11* in cluster 3 were significantly elevated in the year of floral induction (two-way ANOVA, p-value < 0.01; Fig. 4) in addition to *FcNPF1*.*2*. These results from the field transcriptome suggest that there could be a causal relationship between nitrate transporters and flowering in Japanese beech.

**Figure 5:**
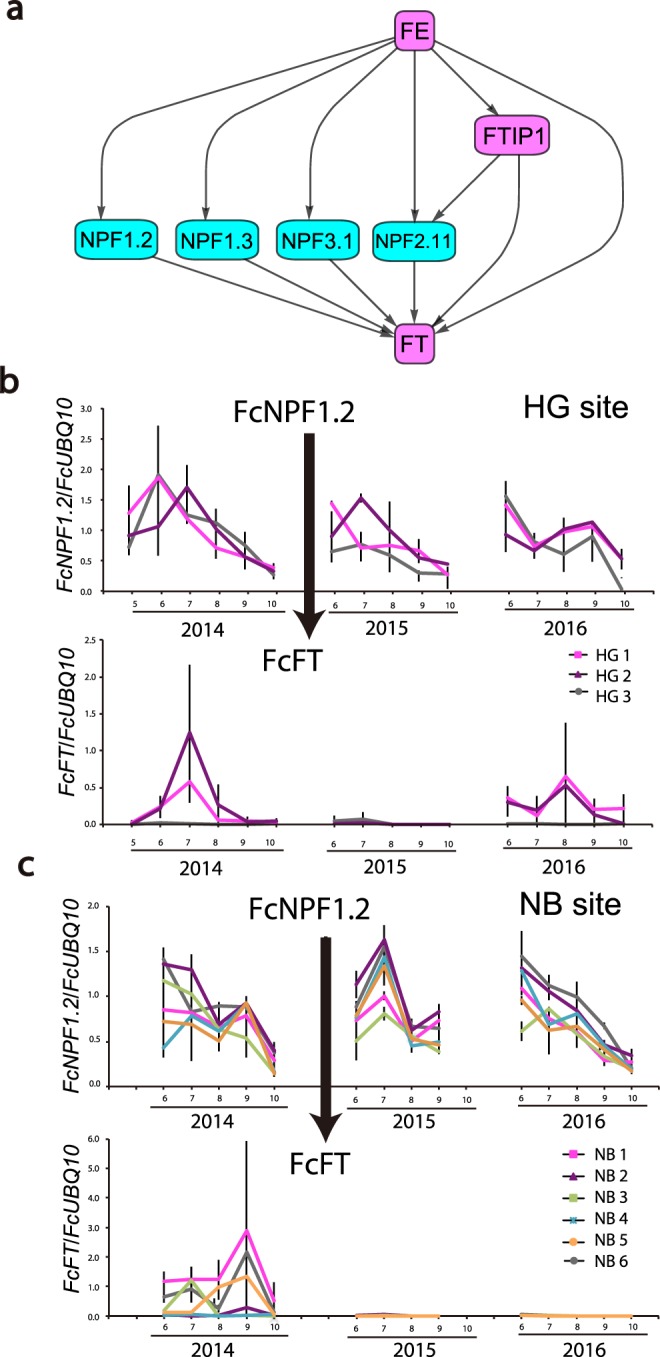
Causal gene regulatory network estimated from the CCM. (**a**) Causal gene regulatory network estimated from the CCM using field transcriptome data. Pink and blue squares represent flowering-time genes and nitrate transporter genes, respectively. Arrows indicate the direction of causal influence. (**b**) Relative expression levels of *FcNFP1*.*2* and *FcFT* (mean ± s.d. of three replicates) of three individuals (HG1–HG3) during 2014–2016 at the HG site. An arrow indicates the direction of causal influence. (**c**) Relative expression levels of *FcNFP2*.*1* and *FcFT* (mean ± s.d. of three replicates) of six individuals (NB1–NB6) during 2014–2016 at the NB site. An arrow indicates the direction of causal influence.

To detect causal relationships between nitrate transporters and flowering-time genes, we performed CCM analysis using time-series data of four nitrate transporters (*FcNPF1*.*2*, *FcNPF1*.*3*, *FcNPF2*.*11*, and *FcNPF3*.*1*) and three flowering-time genes (*FcFT*, *FcFE*, and *FcFTIP1*) that were differentially expressed between years with and without floral induction. We selected the three flowering-time genes because they are known to be expressed in leaf companion cells where nitrate transporter genes are also expressed in *A*. *thaliana*^28,29,38^. The novel causal regulatory relationships detected from the CCM—*FcFT* expression were causally influenced by four nitrate transporters that were in turn upregulated by *FcFE* (Fig. 5a; Supplementary Table S4). Our analysis also showed that *FcFE* regulates the expression of *FcFT* and *FcFTIP1* (Fig. 5a; Supplementary Table S4), which is consistent with previous empirical findings in *A*. *thaliana*^29^. It is important to note that the CCM analysis detects causal patterns, which because of the underlying non-linearity, are not evident from simple comparison of the time series in Fig. 5 (see Introduction).

To confirm the causal influence of nitrate transporters on *FcFT* identified from the analysis of transcriptome data, we generated a longer time series of RT-qPCR data for *FcNPF1*.*2*, which was significantly enriched in the GO analysis (Supplementary Figs S1 and S2), and *FcFT* using leaf samples collected from 2014 to 2016 at both the HG and the NB sites (Fig. 5b,c). Floral induction occurred in both 2014 and 2016 at the HG site (Fig. 1b), while it occurred only in 2014 at the NB site (Fig. 1b). Our RT-qPCR data showed that *FcFT* was activated when floral induction occurred (Fig. 5b,c), justifying the use of *FcFT* expression as a molecular marker of floral induction. At both study sites, CCM using the RT-qPCR data showed significant causality from *FcNPF1*.*2* to *FcFT*, while causality was not detected from *FcFT* to *FcNPF1*.*2* (Supplementary Table S5). These results confirmed the influence of a nitrate transporter on *FcFT*.

## Discussion

**Figure 6:**
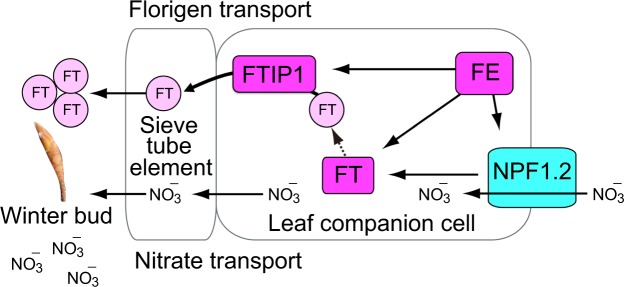
Summary of predicted causal relationships between flowering-time genes and nitrate transporter genes. Florigen and nitrate are simultaneously transported from leaf companion cells to winter bud where floral organs are developed.

Nitrogen has long been known to modify the timing of flowering^39^. In a typical masting species, Japanese beech^14^, previous studies have highlighted the key role of nitrogen in the induction of flowering. Yet, the molecular mechanism involved in this had been remained unknown. Our study provides a new approach combining field transcriptomics and causal inference based on CCM to detect novel causal gene regulatory relationships. CCM is suitable for analyzing our data because resource budget models predicted chaotic flowering dynamics in masting species^12^ and the actual expression patterns of major flowering-time genes revealed significant nonlinearity. From two independent CCM analyses using field transcriptome and qRT-PCR time series data, our study unraveled the causal influence of the nitrate transporter *NPF1*.*2* on *FcFT*, which plays a critical role in floral transition. This gene regulatory relationship is reasonable as the synergistic activation of nitrate transport and florigen synthesis is adaptive to simultaneously satisfy floral transition at the appropriate timing and nitrogen demand needed for flower formation (Fig. 6). Our analysis of field transcriptome data also suggests that *FcFE* could be the upstream regulator of *FcNPF1*.*2* and *FcFT* (Fig. 5a). In *A*. *thaliana*, *FE*/*APL* (*ALTERED PHLOEM DEVELOPMENT*) has been characterized as a regulator of development of vascular tissue^40^. FE is a member of the GARP–CC family^41^ in which several members have been shown to respond to nitrogen or phosphorus deprivation^42,43^. It is tempting to speculate that *FcFE* is involved in the response to nitrate signaling that can be transmitted to signaling pathways of nitrate transport and floral transition in a typical masting species, Japanese beech. Because no direct information about this hypothesis is available, further investigation will be necessary. The recent study reporting that *FcFT* expression patterns cannot be explained by an external environmental factor alone^44^ is consistent with our finding in this study.

Our field transcriptome also revealed the seasonal expression of 23 nitrate transporter genes (21 NPF and 2 NRT2) classified into three different clusters depending on the expression patterns (Fig. 4). The sequential expression of these genes with the seasons may suggest their division of roles to facilitate efficient transportation and utilization of nitrogen under the different seasonal demands in the plant system^45^: nitrogen translocation from roots to shoot in early summer (cluster 1), remobilization from source leaves to sink in the growing season in summer (cluster 2), and nitrogen remobilization during leaf senescence before leaf shedding in the fall (cluster 3). In fact, *FcNPF2*.*3* in cluster 1 and *FcNRT2*.*5* in cluster 3 are orthologs of *NPF2*.*3* and *NRT2*.*5* in *A*. *thaliana* (Supplementary Fig. S2), which are associated with the translocation of nitrogen from roots to shoot^46^ and nitrate loading into the phloem during nitrogen starvation and senescence^47^, respectively. Further clarification and comparison of coordinated expression patterns of nitrate transporters between masting and non-masting species in natural seasonal environments should help to disentangle the complex relationship between nitrogen allocation and reproductive strategies.

Our new approach combining field transcriptomics and causal inference will be a powerful tool to detect novel causal gene regulatory relationships especially in a forest tree species. Here we study a deciduous tree in which transcriptome data from leaves are not available during winter. Performing the same analysis in evergreen trees is needed for evaluating the usefulness of our new approach. In contrast to model plant species such as *A*. *thaliana*, rice, maize, wheat, and tomato, research using forest trees has been difficult because of their long generation times and large genomes, and the lack of well-characterized mutations for reverse-genetic approaches^48^. Nevertheless, given the advent of next-generation sequencing (NGS) technologies, the cost-effective generation of high-throughput sequencing data is increasingly available for both model and non-model species. Moreover, to unravel the molecular mechanisms behind plant responses to naturally fluctuating environments, gene expression analyses in field conditions have been increasingly used in diverse plant species ranging from annual and perennial herbs^49–53^ to trees in the temperate^14^ and tropical zones^13,15^. Our approach taking advantage of this progress in genomics and ecological studies is promising for deciphering the molecular mechanisms crucial for floral transition, development, and environmental responses in natural conditions in a wide range of species.

## Materials and Methods

### Study species and study site

Japanese beech, *Fagus crenata* Blume, is widely distributed from Hokkaido to Kyushu in Japan^54^. It is a typical masting species exhibiting significant variations in flower and seed production between years^55–58^ with high values of the coefficient of variation (1.04–1.79)^58^. Flower initiation occurs in early summer in the year prior to anthesis. Floral and leaf primordia develop in a bud during summer and fall^59^. After winter dormancy, buds break and flowers bloom in spring. Flowers are self-incompatible and wind-pollinated. Seeds develop during summer and fall and drop to the ground before winter^56^. Leaf flushing occurs slightly after flowering. Genome size of *F*. *sylvatica L*., a close relative of *F*. *crenata*, has been estimated as ca. 540 Mbp^60^.

The study site, Hitsuji-ga-oka (HG; 42°59′ N, 141°23′ E; 146.5 m a.s.l.), is situated in Hokkaido, northern Japan (Fig. 1a). Three trees in an arboretum at the HG site were selected. The mean (±SD) height and diameter at breast height (DBH) of three individuals (HG1, HG2, and HG3) were 11.7 m (±2.5) and 36.0 cm (±10.2), respectively. Mean annual precipitation and temperature near the site were 940.5 mm and 7.1 °C, respectively (1981–2010; Meteorological Observation System at the NARO Hokkaido Agricultural Research Center).

To study Japanese beech in natural forests, we also performed sampling at Mt. Naeba (NB; 36°51′ N, 138°46′ E; 900 m a.s.l.). The NB site is located in Niigata Prefecture on Honshu Island, Japan (Fig. 1a), where *F*. *crenata* occupies 92.3% of the basal area (DBH: more than 4.5 cm) of the forest. Six trees were selected from the NB site for the present study. The mean (±SD) height and DBH of six individuals (NB1–NB6) were 21.2 m (±1.5) and 36.4 cm (±6.5), respectively. Mean annual precipitation and temperature during the period 1979–2016 were 2,248 mm and 11.5 °C, respectively, recorded at a nearby meteorological station (36°56′ N, 138°49′ E, 340 m a.s.l., Japan Meteorological Agency). Further details about the NB site are presented elsewhere^45^.

At the HG site, we collected a pair of a leaf and a bud from each of three current-year shoots per tree from May 2014 to October 2016, except during winter (November–May). Samples were taken from the sun-exposed crown (approximately 4 m from the ground) using long pruning shears from 11:30 to 12:30 h. At the NB site, three pairs of leaves and buds were collected from two three-year-old branches in the upper parts of each tree crown (approximately 20 m from the ground) using a mono-ladder with the aid of a 6-m telescopic pruner. Sampling was performed from 09:00 to 10:00 h in 2014 and 2015, and 11:30 to 12:30 in 2016 at the NB site. For each pair of leaf and bud samples, 0.1–0.3 g of leaves and bud tissue were preserved in a 2 ml micro tube containing 1.5 ml of RNA stabilizing reagent (RNAlater; Ambion, Austin, TX, USA) immediately after harvesting. Samples were transferred to the laboratory within 3 hr after sampling and stored at 4 °C overnight and then stored at −20 °C until RNA extraction. During the transport to the laboratory, samples were kept in a cooler box with ice to maintain low temperature. Samples collected from the HG site were used for both transcriptomic and RT-qPCR analyses, while samples collected from the NB site were used only for RT-qPCR.

### Field measurement of flowering intensity

To identify genes that were differentially expressed between years with and without floral induction from the field transcriptome data, we determined the years when floral induction occurred using an index (the proportion of buds which are reproductive) that had been measured previously (Miyazaki *et al*.^14^). Ten branches were selected per individual from the surface of the tree crown, and the status (flower or leaf) of all buds within 1 m of the shoot apex of each branch was classified following the method of Miyazaki *et al*.^14^ in spring from 2014 to 2017 at the HG site. Buds were classified as having a flowering status if they contained female or male flowers. The proportion of buds which are reproductive was calculated as the mean of the flowering rate (number of flowering buds/total number of buds on each branch) over 10 branches. The proportion of buds which are reproductive was used to distinguish floral induction and non-induction years at the individual level. When the proportion of buds which are reproductive was greater than 0.4, the year prior to anthesis was assigned as a floral induction year for the tree because floral induction occurs one year prior to anthesis; otherwise, it was assigned as a non-induction year. Floral induction years were 2014 and 2016 for HG1 and 2 (Supplementary Table S6). There were no floral induction years for HG3 because the flowering intensity was below 0.4 in both years (Supplementary Table S6).

To describe masting dynamics at the NB site, flowering intensity was also measured by the same method as explained above using 3-year-old branches in six individuals used for the RT-qPRC analysis from 2014 to 2017. In 2014, 2016, and 2017, flowering intensity was 0 in all trees, while the mean (±SD) flowering intensity was 0.34 (±0.24; Fig. 1b) in 2015 (Supplementary Table S6).

### RNA extraction

The extraction of total RNA was performed in accordance with the method described by previous study^14^. RNA was extracted independently from leaf and bud samples from three different branches and pooled at each time point. RNA integrity was examined using the Agilent RNA 6000 Nano kit on a 2100 Bioanalyzer (Agilent Technologies), while the RNA yield was determined on a NanoDrop ND-2000 spectrophotometer (Thermo Fisher Scientific).

### Generation of transcriptome next-generation sequencing (NGS) data

We obtained transcriptome data from our samples to design DNA microarray probes. We used 10 samples collected monthly from the individual HG1 at the HG site from June 2013 to October 2014, except during winter (November–May). Five to six micrograms of total RNA extracted from leaf and bud of each sample was sent to Hokkaido System Science (Sapporo, Japan) where a cDNA library was prepared with Illumina TruSeq Sample Prep Kit and paired-end transcriptome sequencing was conducted using the Illumina Hiseq2000 sequencer (Illumina, San Diego, CA, USA) for each sample. Illumina sequence adapters were removed from raw read sequences using cutadapt (Ver. 1.1)^61^. Low-quality bases (Q < 20) were trimmed from the tail of each read with Trimmomatic (Ver. 0.32)^62^. The resulting reads shorter than 50 bp were discarded. “De novo transcriptome assembly” was conducted using Trinity (Ver. 2.0.6)^63,64^.

Quality-controlled reads were mapped to the assembled transcript sequences by bowtie (Ver. 1.1.1, Langmead *et al*., 2000) and the abundance of each transcript was quantified with RSEM (Ver. 0.1.19)^65^. For each pair of monthly data, we detected differentially expressed transcripts using edgeR (ver. 3.8.6)^66^ and obtained a list of 19,996 of them, which was used to design custom microarray slides.

### Probe design for DNA microarray

For custom microarray slides, we used the assembled sequences of the transcripts generated by NGS of *F*. *crenata*. The array was designed using the e-array portal for array design hosted by Agilent (https://earray.chem.agilent.com/earray/). We selected the assembled sequences for array design from two analyses, homology search and identification of differentially expressed transcripts. The first group included 26,951 transcript sequences that showed homology against *Vitis vinifera*, *Populus trichocarpa*, and *A*. *thaliana* as determined by BLASTX searches (e-value cut-off: 10^−10^; http://www.ncbi.nlm.nih.gov/BLAST). The second group included 19,996 sequences for which a greater than twofold expression difference was detected in any potential pair of monthly RNA-seq data from 2015 to 2016. For array probe design, we also added cDNA from three genes, *FcFT*, *FcAP1*, and *FcLFY* (GenBank IDs AB775532, AB674454, and AB775533, respectively), which were previously isolated in *F*. *crenata*^14^. Finally, 61,657 probes were generated from 46,950 transcripts for 8 × 60 K array format.

### Microarray analysis

For each of three individuals at the HG site, RNA was extracted independently from leaf and bud samples from three different branches and pooled at each time point from June 2015 to October 2016. One hundred nanograms of the pooled total RNA extracted from leaf and bud of each sample collected from June 2015 to October 2016 at the HG site was amplified, labeled, and hybridized to a 60 K Agilent 60-mer oligomicroarray, in accordance with the manufacturer’s instructions, for each time point (*n* = 3) based on the one*-*color method. Hybridized microarray slides were scanned by an Agilent scanner. Relative hybridization intensities and background hybridization values were calculated using Agilent Feature Extraction Software (9.5.1.1). After the removal of probes with no signal over all time points, we obtained time-series data of 25,556 independent probes for three individual trees. Each time series was normalized to have a mean of zero and a standard deviation of one.

A total of 153 probes had a unique Blast hit against flowering-time genes of *Arabidopsis thaliana* included in the database FLOR-ID^67^ (Supplementary Table S2). Among these flowering-time genes, functional verification of three genes, *FcFT*, *FcLFY*, and *FcAP1*, was previously performed using transgenic *A*. *thaliana*^14^. To examine the effect of floral induction status and season on gene expression, two-way ANOVA with the error term of tree ID was used. We set two categories for seasons, summer (Jun–Aug) and fall (Sep–Oct). The heat map was drawn from the time series of gene expression levels using Bioconductor (http://www.bioconductor.org) implemented in R software (v3.4). In the heat map, columns of the data were arranged based on unsupervised clustering using Pearson’s correlation coefficient as a distance metric and the average-linkage hierarchical clustering algorithm.

### RT-qPCR analysis

Leaf samples collected during Jun–Oct in 2014–2016 at the two study sites (HG and NB) were used for RT-qPCR analysis. Samples collected in 2015 and 2016 at the HG site were the same as those used for the microarray analysis. RNA extraction and synthesis of cDNA were performed in accordance with the method described by Miyazaki *et al*.^14^. We quantified the expression levels of *FcFT* and *FcNPF1*.*2* using the expression level of *FcUBQ10* as a reference, using the Bio-Rad CFX connect real-time PCR detection system (CRX96 Touch) with SsoFast EvaGreen super mix. Primers used for RT-qPCR are listed in Supplementary Table S7. Previous studies showed that the relative expression level of *FcFT* normalized by *FcUBQ10* is a reliable indicator of the timing and occurrence of floral transition in *F*. *crenata*^14,59^. The mean (±SD) of Ct values of *FcUBQ10* was 25.35 (±0.68).

### Sequence analysis

Sequences of the *NPF-1*.*2* gene were identified using the basic local alignment search tool (BLAST) against the NCBI database (http://www.ncbi.nlm.nih.gov/BLAST/). Sequences were aligned with *NPF-1*.*2* homologs and the phylogeny based on amino acid sequences was analyzed using the neighbor-joining method based on the Jones–Taylor–Thornton model in MEGA7^68–70^.

### Time-series analysis using Empirical Dynamic Model (EDM)

To detect the causal relationship between genes, we applied an equation-free framework of time-series analysis called CCM^21^. CCM is based on Takens’ theorem, which proves that a multidimensional dynamical system can be reconstructed with time-delay embedding of any single time series^71^, as time series preserve the information of their causal variables. In addition, the CCM leverages orbital instability, a characteristic of nonlinearity, to identify significant causality among interacting variables. Thus, only if a variable *X* is causally influencing *Y*, the dynamics of *X* can be recovered by *Y* and this prediction skill can be improved as data points used for prediction become dense^21^. Our analyses proceeded in three steps in accordance with the standard protocol of CCM analysis: (1) we determined the optimal embedding dimension for each time series, (2) tested its nonlinearity, and (3) performed causality tests pairwise for all combinations of time series.

Significant test of causality was performed via the following procedure. First, we calculated the cross-mapping skill (Pearson’s coefficient correlation, *ρ*) with the minimum (=optimal embedding dimension *E* + 1) and maximum library lengths (=observed data points) using 1,000 bootstrap samples (*ρ*_min_ and *ρ*_max_, respectively). Then, we randomly generated 1,000 surrogate (null) data of the putative causal variable, which preserves seasonality and power spectrum, and calculated the prediction skill (*ρ*_surr_) in the direction of the library variable cross-mapping surrogate one. We judged the cross-mapping as significantly causal using two criteria: (1) mean of *ρ*_max_ is higher than both zero and the 95% upper confidence limit of *ρ*_min_, and (2) mean of *ρ*_max_ minus the 95% upper confidence limit of *ρ*_surr_ is larger than *ε*. To test the null hypothesis, we adapted the severer criterion for transcriptome data (24 data points for each tree) that is shorter than the RT-qPCR data (36 data points for each tree) by setting *ε* = 0.02 for the former and 0.01 for the latter because a type-one error is more likely to occur in shorter time series^72^.

We set the gene expression levels from winter to early spring (from November to May) as zero because leaves fell during these seasons in deciduous *F*. *crenata* and used the monthly transcriptome data from January 2015 to December 2016 for the CCM analysis (our data include 24 data points for each of three individuals). To confirm the novel causal relationship between the nitrate transporters and flowering-time genes, we used longer time-series data of *FcNPF1*.*2* and *FcFT* quantified by RT-qPCR from January 2014 to December 2016 for the CCM analysis (36 data points for each tree). Time-series data from different individuals were combined using dew drop regression (Hsieh *et al*., 2008) for both transcriptome and RT-qPCR data. The number of individuals used for our analyses was three and six for the HG and NB sites, respectively. All statistical analyses were conducted using the R software package (R Core Team. *R: A language and environment for statistical computing* R Foundation for Statistical Computing (2014) available at http://www.R-project.org/), and causality analyses were conducted using the “rEDM” package in R.

## Supplementary information

revised SI

Supplementary Dataset 1

Supplementary Dataset 2

Supplementary Dataset 3

Supplementary Dataset 4

Supplementary Dataset 5

Supplementary Dataset 6

## Data Availability

The sequence data and DNA microarray data that support the findings of this study are available from the NCBI Shotgun Assembly Sequence Database (TSA) (accession GHGX00000000) and NCBI GEO database (accession GSE126540).
