# Supplementary material for: Field transcriptome revealed a novel relationship between nitrate transport and flowering in Japanese beech
Source: Sci Rep. 2019 Mar 13;9:4325. doi: 10.1038/s41598-019-39608-1 (PMC6416253; doi:10.1038/s41598-019-39608-1)
Supplement: Supplementary file 1 — revised SI [file 41598_2019_39608_MOESM1_ESM.pdf]

Supplementary Materials for
Field transcriptome revealed a novel relationship between nitrate transport and flowering in Japanese beech

Akiko Satake, Kazutaka Kawatsu, Kosuke Teshima, Daisuke Kabeya, Qingmin Han

This supplementary material includes following materials.

Supplementary Figure S1
Supplementary Figure S2
Supplementary Figure S3
Supplementary Table S7

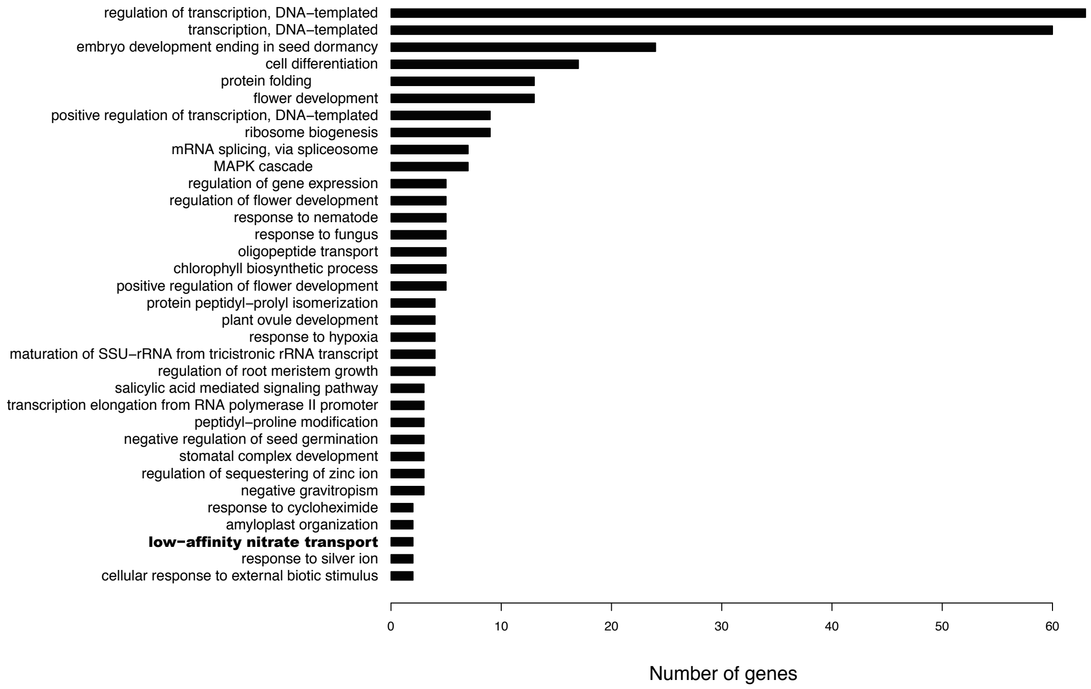

Supplementary Figure S1 | GO enrichment of differentially expressed genes (DEGs) in biological processes according to DAVID functional annotation. Significantly enriched biological processes of DEGs depending on the status of floral induction.

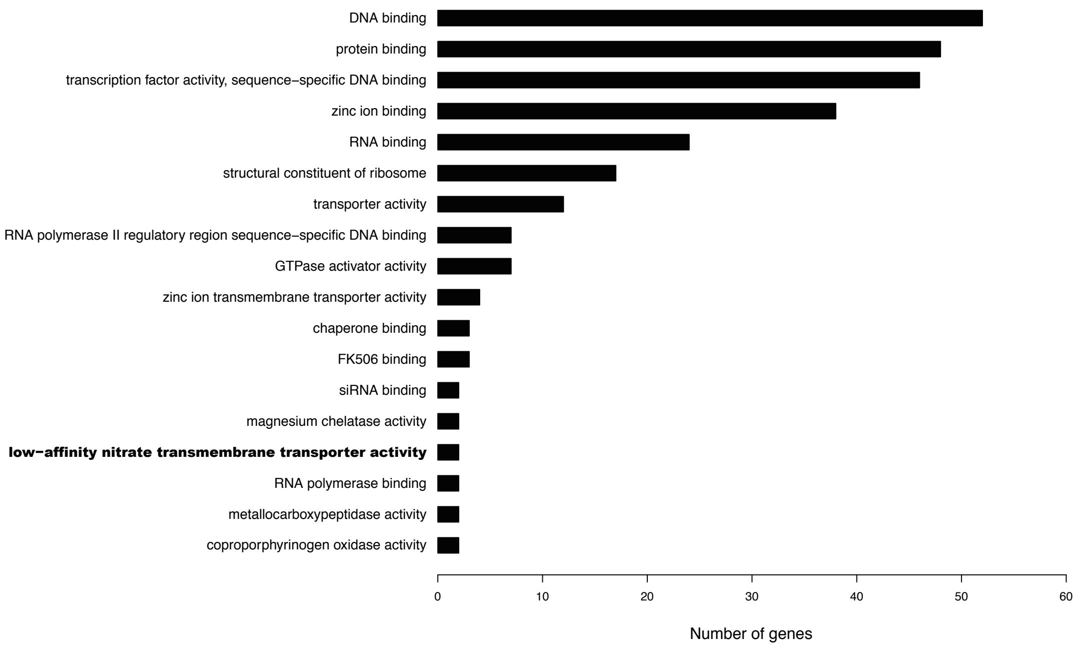

Supplementary Figure S2 | GO enrichment of differentially expressed genes (DEGs) in molecular functions according to DAVID functional annotation. Significantly enriched molecular functions of DEGs depending on the status of floral induction.

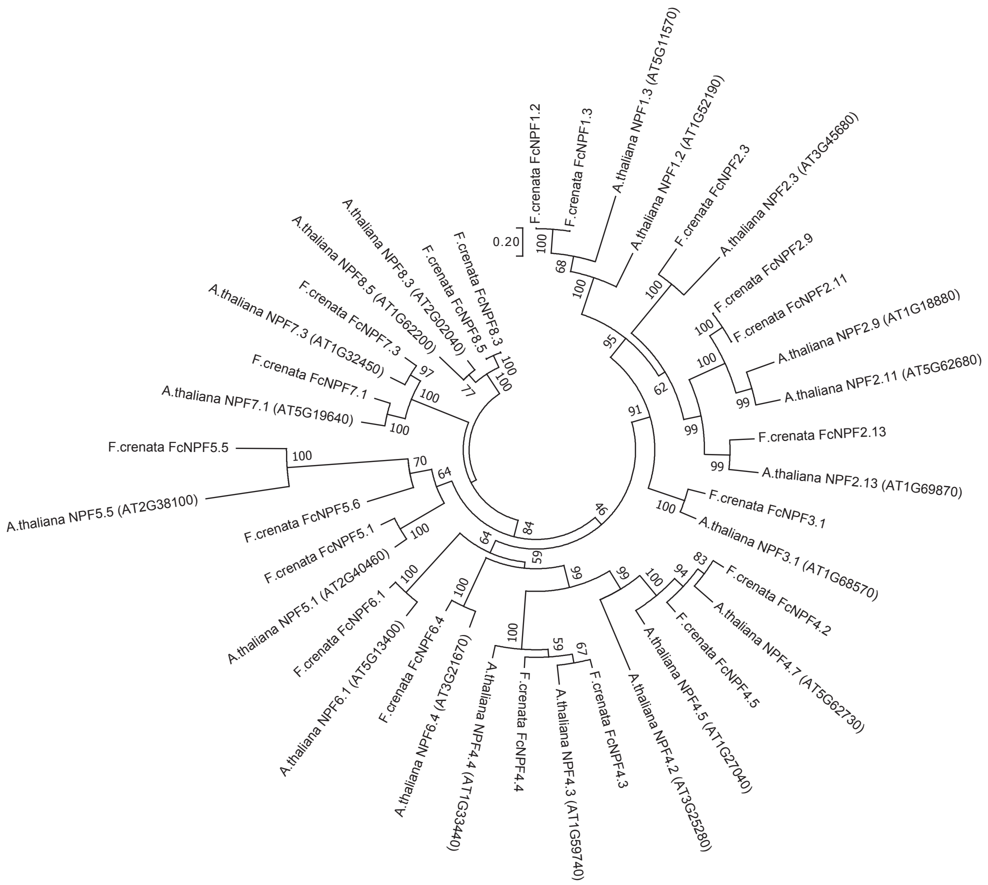

Supplementary Figure S3 | Phylogenetic relationship of NPF proteins in *F. crenata* and *A. thaliana.* The phylogenetic tree was created using using the Maximum Likelihood method based of the JTT matrix-based model in MEGA7. The evolutionary distances were computed using the JTT matrix-based method. The rate variation among sites was modeled with a gamma distribution (shape parameter = 1). The numbers at the branches are the bootstrap values (1,000 replications).

Supplementary Table S7 | List of primers used for qRT-PCR in this study.

Genes	Reference	Sequence
FcFT	Miyazaki et al. (2014)	5'- GCCAGTGGCTGCAGTTTATT -3'
		5'- GAGTTTTTCTCTCATCGCCTTC -3'
FcNPF1.2	—	5'- CAAGCAACATCAATAAGGCTCAC -3'
		5'- ACCCATCTCCTTCCTCTAAAACC -3'
FcUBQ10	Miyazaki et al. (2014)	5'- TTGTGGTTCCATGGTCGAGT -3'
		5'- CGATGCGGCAGGTAAACTAA -3'
